# Adequate timing and constant supervision are the keys for successful implementation of levothyroxine or levothyroxine/paracetamol absorption test

**DOI:** 10.1186/s13044-020-00079-6

**Published:** 2020-05-18

**Authors:** Krzysztof C. Lewandowski, Katarzyna Dąbrowska, Magdalena Basińska-Lewandowska, Marek Bolanowski, Marek Ruchała, Andrzej Lewiński

**Affiliations:** 1 Department of Endocrinology and Metabolic Diseases, Medical University of Lodz, Rzgowska 281/289, 93-338 Lodz, Poland; 2 Department of Endocrinology and Metabolic Diseases, Polish Mother’s Memorial Hospital - Research Institute, Lodz, Poland; 3 Department of Endocrinology, Diabetes and Isotope Therapy, Medical University of Wroclaw, Wrocław, Poland; 4 Department of Endocrinology and Metabolic Diseases, Medical University of Poznan, Poznan, Poland

**Keywords:** Levothyroxine, Paracetamol, Acetaminophen, Absorption test, Pseudomalabsorption, Non-adherence, Non-compliance

## Abstract

**Background:**

Levothyroxine (LT_4_) pseudomalabsorption due to medication non-adherence results in significant costs for Health Service. High dose LT_4_ or LT_4_/paracetamol absorption test is used in such cases. Hence, establishment of an optimal test protocol and timing of sample collection is of utmost importance.

**Case presentation:**

A 34-year old woman was admitted to our Department because of severe hypothyroidism [on admission thyrotropin (TSH) > 100 μIU/ml, free thyroxine (FT_4_) 0.13 ng/dl (ref. range 0.93–1.7)] despite apparently taking 1000 μg of LT_4_ a day. Autoimmune hypothyroidism had been diagnosed 4 years before during post-partum thyroiditis. Subsequently, it was not possible to control her hypothyroidism despite several admissions to two University Hospitals and despite vehement denial of compliance problems. There was no evidence of coeliac disease or other malabsorption problems, though gluten-free and lactose-free diet was empirically instigated without success. A combined paracetamol (1000 mg)/LT_4_ (1000 μg) absorption test was performed in one of these Hospitals. This showed good paracetamol absorption (from < 2 μg/ml to 14.11 μg/ml at 120 min), with inadequate LT_4_ absorption (FT_4_ increase from 5.95 pmol/l to 9.92 pmol/l at 0 and 120 min respectively). About 2 years prior to admission to our Department the patient was treated with escalating doses of levothyroxine [up to 3000 μg of T_4_ and 40 μg of triiodothyronine (T_3_) daily] without significant impact on TSH (still > 75 μIU/ml, and FT_4_ still below reference range).

After admission to our Department we performed a 2500 μg LT_4_ absorption test with controlled ingestion of crushed tablets, strict patient monitoring and sampling at 30 min intervals. We observed a quick and striking increase in FT_4_ from 0.13 to 0.46, 1.78, 3.05 and 3.81 ng/dl, at 0, 30, 60, 90 and 120 min, respectively. Her TSH concentration decreased to 13.77 μIU/ml within 4 days. When informed, that we had managed to “overcome” her absorption problems, she discharged herself against medical advice and declined psychiatric consultation.

**Conclusions:**

Adequate patient supervision and frequent sampling (e.g. every 30 min for 210 min) is the key for successful implementation of LT_4_ absorption test. Paracetamol coadministration appears superfluous in such cases.

## Background

Levothyroxine (LT_4_) pseudomalabsorption due to poor adherence, or non-adherence (also termed non-compliance) to prescribed regimen constitutes a rare but serious problem, given the fact that genuine cause of the problem is often denied by patient. Furthermore, such cases are also characterised by poor attendance for follow-up appointments, by some patients, once poor-adherence to medication is mentioned [1]. In cases of LT_4_ non-adherence, a high dose LT_4_ absorption test is often used [1–3]. Levothyroxine absorption test is also used in cases of suspected interference in TSH and/or free thyroid hormone measurements [4, 5].

Levothyroxine absorption test is, however, not standardised, both in terms of optimal timing of sampling, as well as in terms of potential utility of co-administration of paracetamol (acetaminophen), as suggested by some authors [6]. Hereby we present a case of 34-year old female patient with LT_4_ pseudomalabsorption due to non-adherence to prescribed therapy with a history of multiple admissions to two academic units and two previous LT_4_ absorption tests that had lead to misleading results leading to a recommendation of treatment with massive doses of LT_4_ (3000 μg/day).

## Case presentation

A 34 year old woman (height 164 cm, weight 57 kg, BMI 21.2 kg/m^2^) was referred for investigations in our Department following a dramatic plea from her General Practitioner (GP) addressed to Chief Endocrine Consultant for Poland (AL). Her GP explained that despite treatment with high doses of LT_4_ and multiple admissions to two University Departments of Endocrinology, as well as to local District General Hospital, her TSH concentrations oscillated between 300 and 500 μIU/ml (ref. 0.27–4.2) with very low free thyroxine (FT_4_) concentrations. In GP opinion, advice received so far had not provided her with “*any successful treatment plan*”. GP also inquired whether administration of intravenous preparations of LT_4_ would be appropriate in her case. Copies of her previous extensive medical records were enclosed. Hospital admission was organised and the patient was admitted under the care of medical team with particular experience in cases of LT_4_ malabsorption, as well as assay interference (KCL, KD).

**Fig. 1:**
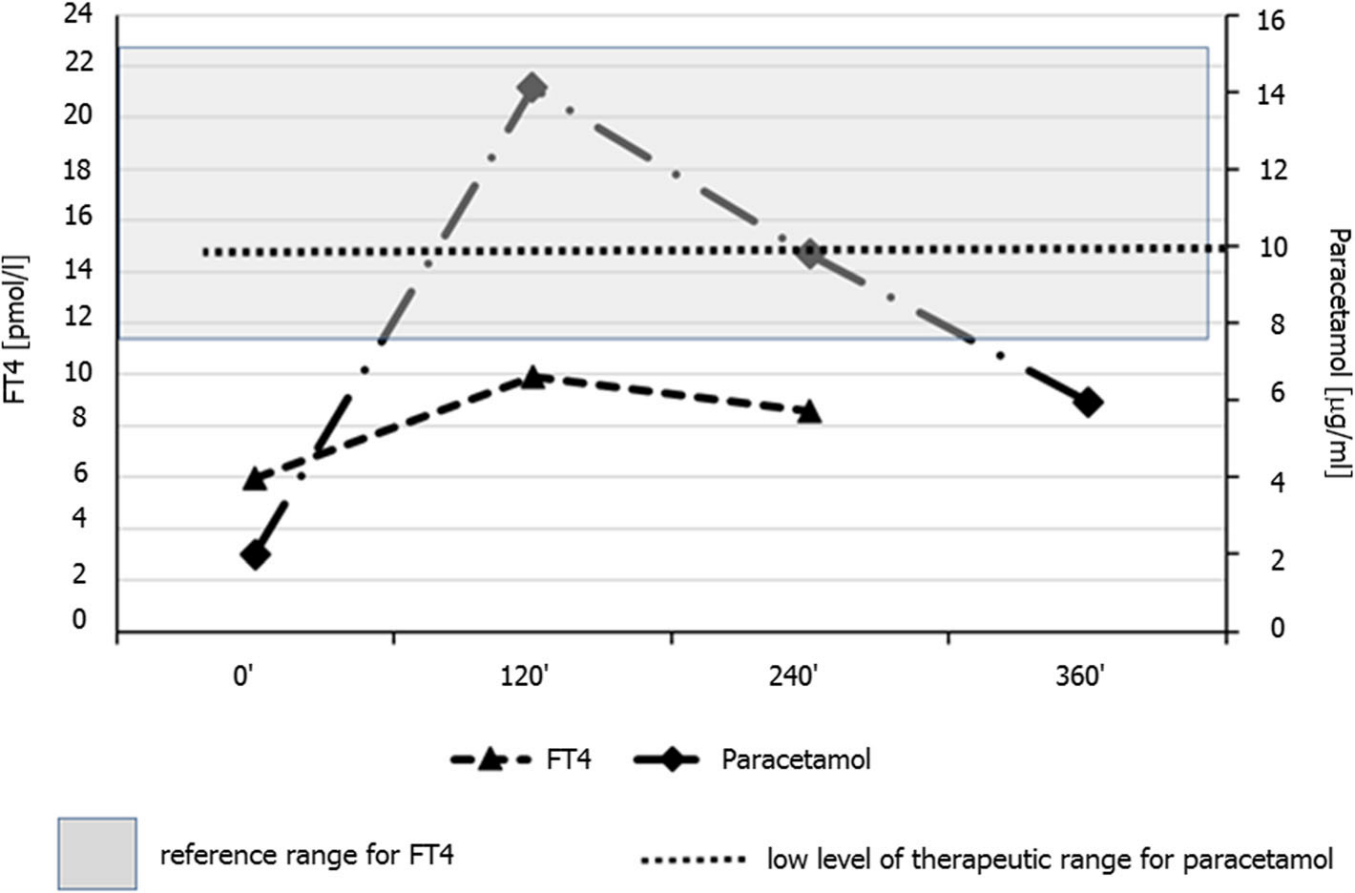
Results of combined levothyroxine (1000 μg) and paracetamol (1000 mg) oral absorption test in a female patient with suspected levothyroxine malabsorption about 2 years prior to admission to our Department. Darker area represents reference range for free T_4_ (11.5–22.7 pmol/l)

Patient history and review of available documentation revealed that she had been well till about 4 years before (then aged 30), where she developed autoimmune hypothyroidism as post-partum thyroiditis. She denied any history of post-partum depression, while early clinic documentations around the time of diagnosis was unavailable. Approximately 1 year later she developed problems with uncontrolled hypothyroidism despite increasing doses of LT_4_. Approximately 2 years after diagnosis she was admitted to Department of Endocrinology of another University Hospital. Her weight was 54 kg and there was no obvious evidence of malabsorption or other hormonal problems. Her TSH, however, was above 75 μIU/ml, and she remained profoundly hypothyroid, so a combined LT_4_ (1000 μg)/paracetamol (1000 mg - type of preparation not specified) absorption test was performed. This showed rather weak absorption of LT_4_ (about 66% increase in FT_4_ concentration), but quite good absorption of paracetamol (where the therapeutic target level of paracetamol was assumed to be 10–20 mg/l, i.e. 10–20 μg/ml) [7] (see Fig. 1), at some point she also had 500 μg LT4 absorption test (first sample after 120 min) that showed only 20% increase in FT_4._

Based on the result of this test the managing team concluded that some genuine malabsorption problems must be present.

As there was also a borderline concentration of anti-gliadin antibodies (titres of other coeliac disease-related antibodies were normal), she was referred for further investigations in the Department of Gastroenterology, where no obvious abnormalities, apart from tendency to constipation, were found. It should be noted, however, that she declined gastroscopy, as she insisted on the test being performed under general anaesthesia that according to her managing team “*was not possible in view of her profound hypothyroidism*”. Despite the lack of any clear evidence of coeliac disease, an empirical trial of both gluten-free and lactose-free diet was instigated.

This was followed by an attempt to “overcome” her presumed malabsorption with massive doses of LT_4_, as well as combined LT_4_/T_3_ medication (Novothyral®) administered as an inpatient over a 24 day period. Following trial of various combinations of medications (Table 1), with doses of LT_4_ reaching an enormous 3000 μg/day she achieved an increase of free T_3_ (FT_3_) from 3.06 to 4.19 pmol/l (ref. Range 2.76–6.45), however, without any measurable impact on TSH concentrations and only a 20% increase in FT_4_.

**Table 1:** Thyroid hormone concentrations following administration of various combinations of massive doses of levothyroxine and triiodothyronine in order to “overcome” presumed malabsorption of levothyroxine

Thyroid function tests (07.2017)					
Date	Time	TSH [μIU/ml] [Ref. Range 0.4–4.0]	FT_**4**_ [pmol/l] [Ref. Range 11.5–22.7]	FT_**3**_ [pmol/l] [Ref. Range 2.76–6.45]	Daily dose p.o.
**Day 1**	10:00	> 75	4.12	3.06	LT_4_ 1700 μg and T_3_ 40 μg
	18:00	nm*	3.99	3.16	
	2:00	nm	4.83	2.95	
**Day 2**	10:00	nm	4.67	3.27	LT_4_ 1700 μg and T_3_ 40 μg
	18:00	nm	4.35	3.24	
	2:00	nm	4.59	3.01	
**Day 3**	18:00	nm	4.92	2.93	LT_4_ 2200 μg and T_3_ 40 μg
	2:00	nm	4.38	2.87	
**Day 4**	8:00	nm	6.38	2.86	LT_4_ 3200 μg and T_3_ 40 μg
	18:00	nm	5.73	3.29	
	2:00	nm	5.78	3.55	
**Day 5**	8:00	nm	5.96	2.93	LT_4_ 3200 μg and T_3_ 40 μg
**Day 6**	8:00	nm	6.64	3.9	LT_4_ 3200 μg and T_3_ 40 μg
**Day 7**	8:00	nm	6.96	3.62	LT_4_ 3200 μg and T_3_ 40 μg
**Day 15**	8:00	nm	6.62	3.81	LT_4_ 1700 μg and T_3_ 40 μg
**Day 24**	8:00	> 75	5.53	4.19	LT_4_ 3000 μg

*nm = not measured

On admission to our Department the patient was biochemically profoundly hypothyroid [TSH > 100 μIU/ml, FT_4_ 0.13 ng/dl (ref. range 0.93–1.7)] despite apparently taking 1000 μg of LT_4_ a day. Her skin was dry and ankle reflexes were markedly delayed. She seemed, however, to be very well adopted to her hypothyroid state, blood pressure was 116/74 mmHg, heart rate 70 beats/minute, with a history of only 3 kg weight gain over 2 year period. Her periods were heavy and mildly irregular (every 34–45 days). Interestingly, no third party was authorised by the patient to be informed about her medical condition, even though she admitted that she was living with a partner. As an initial part of investigation we performed serial TSH dilution test, in order to rule out possible assay interference (Table 2). This is the standard procedure to demonstrate linearity of hormone concentrations following serial dilutions [8].

**Table 2:** Results of TSH dilution test performed on a day prior to LT4 absorption test

TSH sample dilution	TSH [μIU/ml]	TSH [μIU/ml] after recalculation
No dilution	289.3	289.3
1:4	72.8	291.2
1:16	19.7	315.2
1.32	10.1	323.2

Taking into account that she had been apparently taking 1000 μg of LT_4_ a day, a 2500 μg (i.e. 2.5 times her presumed daily dose) LT_4_ absorption tests was performed. She received crushed tablets of standard LT_4_ preparation (Euthyrox N®), with a lactose-containing base. The test was, however, performed under strict supervision. In particular, the patient was told to remain for an entire duration of the test in the room, where endocrine dynamic tests are performed in our Department. Blood samples were taken every 30 min. Several nurses and other patients undergoing dynamic tests of pituitary function (such as glucagon stimulation test) were also present in the same room.

**Fig. 2:**
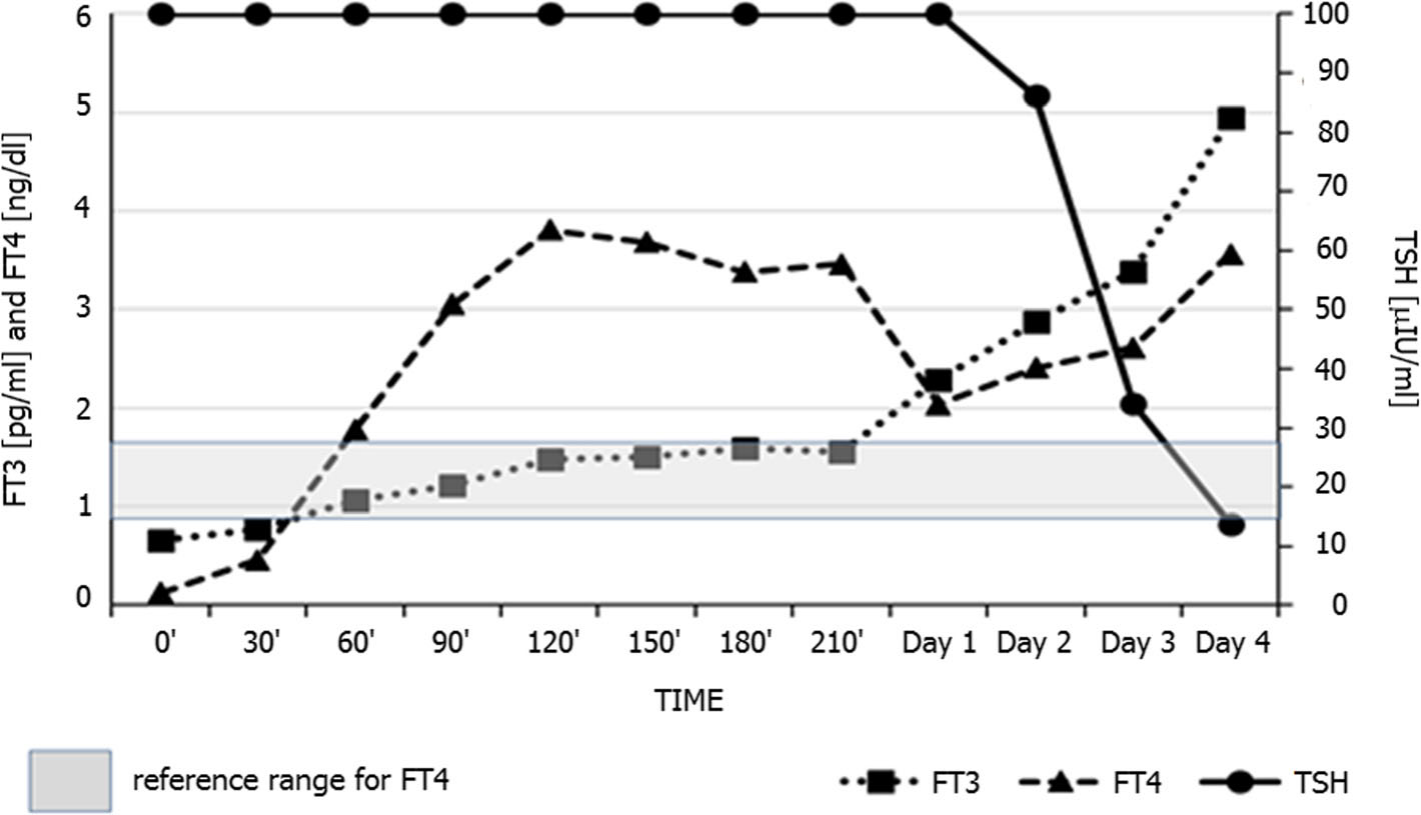
Results of oral 2500 μg levothyroxine absorption test in a female patient with suspected levothyroxine malabsorption performed in our Department under strict supervision. Darker area represents reference range for free T_4_ for our assay (0.93–1.70 ng/dl). Avid absorption of levothyroxine is clearly demonstrated followed by near normalization of TSH concentrations after 4 days

Under these circumstances, LT_4_ absorption test revealed an excellent absorption of LT_4_, with FT_4_ concentrations surpassing upper limit of FT_4_ reference range at 60 min and reaching concentrations about 25 times above the baseline FT_4_ concentration at 120 min of the test (Fig. 2). This was followed by a marked decrease and near normalisation of TSH concentration after 4 days.

As we were not convinced that general anaesthesia was indeed necessary for gastroscopy in the setting of hypothyroidism, and in view of clinical anaemia (haemoglobin 8.1 g/dl) with iron deficiency, standard gastroscopy with duodenal biopsies was performed. This showed evidence of Helicobacter pylori infection, but failed to demonstrate any histological evidence of villous atrophy (grade “nil” on Marsh scale of duodenal atrophy) [9].

The patient was subsequently informed that we had managed to overcome her “absorption problems”. In such circumstances she discharged herself against medical advice. She declined any psychological or psychiatric consultation. We suggested LT_4_ (Euthyrox N®) 200 μg/day (still a high dose of about 3.5 μg/kg of body weight), as well as Helicobacter pylori eradication course, with an outpatient check-up of thyroid function after 4–5 weeks. We also suggested a possibility of supervised weekly ingestion of 1400 μg of LT_4_ once a week, with possible further decrease of the dose to about 1.6 μg/kg/day (~ 90–100 μg/day) in the outpatient setting, depending on her TSH and fT4 concentrations.

## Discussion

Our study demonstrates a case of severe and prolonged LT_4_ pseudomalabsorption due to do non-compliance linked with manipulative behaviour. This psychiatric disorder, known as factitious disorder, is characterised by lying, sometimes involving the use of aliases, multiple hospital admissions without specific reasons, and extensive knowledge of medical symptomatology [10, 11]. The purpose of such behaviour is to adopt the “sick role” that may be associated with some form of gratification, e.g. release from daily duties, centred attention of family members, etc. A psycho-analytic explanation formulated by O’Shea [12] suggests that feeling of neglect and being abandoned are the underlying reasons for such behaviour.

In such cases, if associated with non-adherence to LT_4_ treatment, a LT_4_ absorption test may be used [1, 2, 11, 13]. It is documented that peak concentrations of FT_4_ are typically observed about 120 min after test dose [2, 11, 13, 14], though data of Ching Sun et al. [3] suggest that almost peak concentrations of FT_4_ may be observed as soon as between 60 and 90 min after the test dose. It is not entirely clear what extent of FT4 increase should be considered an adequate response during LT4 absorption test. Soares et al. [15] suggest that an increase in FT4 concentration should be at least 2.5 times above the baseline concentration as long as the initial FT4 concentration is below the lower limit of reference. Absorption of LT4 can be further enhanced if the tablets are administered in a crushed form [16].

In our case, we have documented, however, an excellent absorption of LT_4_ despite evidence of rather poor absorption during previous LT_4_ absorption tests performed in other institutions. In one of these tests quite good absorption of paracetamol was observed despite rather inadequate absorption of LT_4_ (Fig. 1). This had lead supervising physicians to reach an erroneous conclusion that the patient was suffering from a genuine malabsorption despite the lack of evidence of coeliac or other gastrointestinal diseases. In consequence, she had been put on both gluten and lactose-free diet that was followed by rather unsuccessful attempts to overcame presumed LT_4_ malabsorption with massive LT_4_ (and T_3_ doses) (Table 1**)**. Prolonged ingestion of such high doses of LT_4_ might have been potentially very dangerous, if the patient had suddenly decided to follow the prescribed regimen.

It should be noted that paracetamol absorption test has widely been validated as a marker of gastric emptying [17], while coadministration of LT_4_ and paracetamol (i.e. a combined LT_4_/paracetamol absorption test) was suggested as a good method to check for LT_4_ malabsorption [6]. It is not clear what kind of paracetamol formulation was administered during the test performed in our patient, but it is known that absorption of paracetamol is much faster if administered in a crushed form or together with bicarbonate. In case of paracetamol the time for maximal post-dose concentration (t_max_) is about 1.58 h for standard tablets, but it decreases to as much as 0.42 h (about 25 min), if co-administered with bicarbonate [18]. Indeed the study of Sanaka et al. [19] suggests that assessment of paracetamol concentrations (if given in a liquid form) can be performed as soon as after 15 and 30 min in order to reliably estimate gastric emptying.

In our case, the first assessment of FT_4_ and paracetamol concentrations were performed rather late, i.e. 2 h after ingestion of test doses. In our opinion, such timing impeded adequate supervision of the patient during the crucial absorption period (i.e. the first hour after tablet ingestion). In such cases a possibility of a manipulative behaviour must be always suspected. Notably, in a study by Thynne and Doogue [6] sampling was performed at hourly intervals during a four-hour period.

In our case there is a strong suspicion that the patient had not been supervised correctly, i.e. she was likely to swallow the tablets in a supervised manner, but must have been subsequently left unsupervised till the first blood sampling (i.e. after 120 min). There is a possibility that if subsequently she had managed to provoke vomiting, e.g. 20–25 min after the test dose, then most of the paracetamol dose might have been already absorbed, in contrast to LT_4_ that is absorbed more slowly. This, in turn could result in misleading test result.

In contrast, in our Institution we have observed a very rapid absorption of LT_4_, when the patient was prevented from leaving the area, where blood samples were being collected at 30 min intervals. She was instructed that all patients who require frequent blood testing, e.g. during dynamic tests for pituitary function, had to remain in the nurse-supervised Endocrine Dynamic Test Room for the entire duration of the test. Importantly, she was aware that such procedure was not “designed” particularly for her, as other patients were also present. As a result of such approach, we have managed to ensure an adequate patient supervision and clearly demonstrated normal absorption of LT_4_. In such circumstances we have also demonstrated that there is no need to co-administer paracetamol. Furthermore, paracetamol absorption test without adequate supervision may be indeed misleading. In our case, though the patient discharged herself against medical advice, the diagnosis of deliberate non-compliance with medication regimen was established and her General Practitioner was informed about the outcome of our investigations. Furthermore, we offered a possibility of a supervised ingestion of her LT_4_ dose once a week. Such an approach was found to be safe and validated by several authors [20–22].

## Conclusion

In summary, we have demonstrated that adequate supervision and frequent sampling is the key for successful implementation of LT_4_ absorption test in manipulative patients with deliberate medication non-adherence. Such an approach should obviate the need for several readmissions associated with enourmous costs for health service and multitude of unnecessary investigations. Furthermore, adequate supervision and frequent sampling during initial part of the test renders paracetamol coadministration superfluous.

## Data Availability

Data availability from the authors on request.

## Abbreviations

FT_3_: Free triiodothyronine
FT_4_: Free thyroxine
LT_4_: Levothyroxine
T_3_: Triiodothyronine
TSH: Thyroid stimulating hormone
